# Parental care status and sexual risk behavior in five nationally-representative surveys of sub-Saharan African nations

**DOI:** 10.1186/s12889-021-12437-6

**Published:** 2022-01-10

**Authors:** Sarah Elizabeth Neville, Indrani Saran, Thomas M. Crea

**Affiliations:** Boston College School of Social Work, 140 Commonwealth Avenue, Chestnut Hill, MA 02467 USA

**Keywords:** Orphans, Sub-Saharan Africa, Alternative care, Sexual risk taking, Adolescence

## Abstract

**Background:**

About 10% of children worldwide do not live with either of their biological parents, and although some of these children are orphans, many have living parents. While research shows that orphaned children in Sub-Saharan Africa tend to engage in more sexual risk behaviors than their peers, possibly due to decreased parental oversight and support, it is unclear if these effects also apply to children separated from their living parents. Exploring the question of whether living without parents, regardless of whether they are deceased, is linked to greater sexual risk-taking, this study is the first, to our knowledge, to examine correlates of parental care status in a multi-country, nationally-representative analysis.

**Methods:**

This study was a secondary analysis of the Centers for Disease Control and Prevention’s Violence Against Children Surveys from Kenya, Malawi, Tanzania, Nigeria, and Zambia. We conducted logistic regressions on *N =* 6770 surveys of youth aged 13 to 17 years to determine if living with their biological parents predicted the odds of engaging in risky sexual behavior, controlling for demographic factors including orphanhood. Post-hoc regressions examined specific risk behaviors.

**Results:**

Compared to those living with both parents, youth not living with either parent had heightened odds of engaging in any sexual risk behavior, even when controlling for orphanhood (*OR =* 2.56, 95% CI: [1.96, 3.33]). Non-parental care predicted heightened odds of non-condom use (*OR =* 3.35, 95% CI: [2.38, 4.72]), early sexual debut (*OR =* 1.80, 95% CI: [1.31, 2.46]), and more sexual partners (*β =* .60, *p <* .001).

**Conclusions:**

This study extends prior research linking orphanhood and sexual risk behavior, lending credence to the idea that it is not parental death, but rather parental absence, that leads to sexual risk in youth. Public health programming in Sub-Saharan Africa should consider targeting not only “orphaned youth,” but all children separated from their parents.

## Background

A large body of research, born out of the global push to intervene for HIV-affected children in Sub-Saharan Africa (SSA), has demonstrated that single orphans (children who have lost one parent) and double orphans (children who have lost both parents) have diminished well-being compared to their peers with two parents [1–6]. For example, one well-established disparity is that orphans are at risk of not being enrolled in school [4, 6]. Notably, this difference persists even when controlling for poverty, and much of it can be explained by children’s living arrangements [7]: the degree of biological closeness between caregiver and child predicts school enrollment [7], and non-orphaned children living with someone other than their biological parents fare more poorly in education and health than children living with both parents [4]. These findings, in sum, suggest that whether or not children live with their parents (i.e., “parental care status”) could be an important predictor of well-being.

In SSA, generally speaking, strong familial and communal bonds traditionally formed the basis of systems of care for children without parental care, and informal fostering by aunts, uncles, grandparents, and other kin is the most common alternative to care by biological parents [8]. Indeed, globally, about 10% of children aged 0 to 14 years reside with neither of their biological parents [9]. Some studies have linked non-parental care with adverse outcomes in young people in SSA. For example, children across SSA who live with grandparents have been found to be substantially more likely to live in poverty than children in other care arrangements [10]. In Rwanda, when controlling for HIV status, children living with individuals who were not their parents had greater depression, anxiety, and irritability than children in parental care [11]. In Zimbabwe, orphaned children living in family-based care were less likely to attend school or have birth registrations compared to non-orphaned children [12]. In a study of five countries including Ethiopia, Tanzania, and Kenya, children in non-parental care experienced more types of traumatic events than those living with a biological parent, and children who lived with two biological parents experienced fewer types of traumatic events than other children [13].

Among youth in SSA, sexual risk behaviors have been shown to be major drivers of HIV/AIDS [14–17]. There is a well-established relationship between parental influence and adolescent sexual risk behaviors, especially in Western samples [18, 19]. However, few recent studies have examined the link between parental care status and youths’ sexual risk-taking, particularly in SSA. A notable exception is a Ghanaian study, which found young people living with both biological parents had a lower risk of having had sexual intercourse, compared with youth living alone, with grandparents, or in other households; levels of parental monitoring explained part of these differences [20].

Though few studies looked at parental care status and sexual risk taking, a large body of literature on orphanhood may lend insights into their potential connection. Children who are orphans are consistently more likely to engage in sexual risk behaviors and to report younger age of sexual debut [21]. In South Africa, youth with at least one deceased parent were significantly more likely to have engaged in sex as compared to non-orphans; among sexually active youth, orphans also reported younger age of sexual debut [22]. Similarly, in Zimbabwe, youth with deceased mothers (i.e., maternal orphans) were more likely to have had sex [23]. This phenomenon is especially well-documented amongst girls. A multi-country study of girls linked orphanhood with early sexual debut in Côte d’Ivoire, Lesotho, Mozambique, and Tanzania [24]. One study found that in Burkina Faso, Ghana, and Malawi, female double orphans had earlier sexual debut than non-orphans, while male double orphans did not [25]. In Zimbabwe, girls with deceased mothers had earlier sexual debut than non-orphaned girls [26]. Gender differences were also found in South Africa, where girl orphans were more likely to have had sex and to have multiple sexual partners than non-orphans, while boy orphans were more likely to have had sex or to have had sex without a condom than non-orphans [27].

Some evidence suggests that parental absence, rather than parental death per se, leads these youth to engage in sexual risk behavior. Investigating the reasons behind orphans’ heightened sexual risk, a study of Ugandan youth with at least one deceased parent found a large portion of youth’s reduction in permissive attitudes towards sexual risk could be explained by youth’s perceived support from caregivers [28]. In a qualitative study of western Kenya, community members explained that orphans and non-orphans engaged in risky sexual behaviors in circumstances where they could not be closely monitored by their parents [29]. They also described gendered effects, stating that among orphaned youth, discrimination against girls drives them towards risky sexual behaviors because they feel undervalued in their homes compared to boys [29]. Perhaps similar dynamics also drive children outside parental care towards sexual risk behaviors; thus, we hypothesize that children without parental care engage in riskier sexual behavior.

Given the importance of sexual risk behaviors to public health, to explore this hypothesis, and responding to the call for researchers to better mine public datasets for information about children’s living situations in the Global South [9], we examine the association between parental care status and sexual risk behavior in youth in five SSA countries. To our knowledge, this is the first study to use nationally-representative data to examine correlates of parental care status.

## Methods

This study was a secondary analysis of the cross-sectional Centers for Disease Control and Prevention’s (CDC) Violence Against Children Surveys (VACS) [30]. VACS are nationally-representative population-based household surveys of 13- to 24-year-olds that measure childhood violence and risk and protective factors of violence. We used five surveys conducted in SSA: Kenya (collected in 2010), Malawi (2013), Tanzania (2009), Nigeria (2014), and Zambia (2014). All surveys used multi-stage sampling designs that included random selection of a regional cluster, probability systematic sampling of households within clusters, and random selection of a respondent from within the household. We included all VACS from SSA publicly available at the time, except the Swaziland VACS, which only sampled girls [31]. For our analysis, we excluded respondents aged 18 years and older. The Kenya dataset included *n* = 1292 observations of 13- to 17-year-olds, Malawi included *n* = 1070, Nigeria *n* = 1840, Tanzania *n* = 1783, and Zambia *n* = 785. All VACS protocols are approved by both the CDC Institutional Review Board (IRB) and ethical review bodies in each host country [30]. This study was granted an exemption from the Boston College IRB.

### Measures

#### HIV sexual risk behavior

We created a composite binary measure to represent whether the respondent had engaged in any sexual risk behaviors (lack of condom use, multiple sexual partners, and early sexual debut). It was coded as 0 if the three binary variables of sexual risk taking were all 0, and as 1 if any of them were 1. If any of the three were missing, it was coded as missing (1.6% missing).

##### Lack of condom use

If the respondent reported engaging in any sex without a condom within the past 12 months, this binary variable was coded as 1, and otherwise as 0 (< 0.01% missing).

There were slight variations in the structure of condom-related survey questions across countries. In Tanzania and Kenya, the VACS asked how often respondents used condoms in the past 12 months with partners they “knew very well,” and then asked the same about partners they “did not know very well.” On the other hand, in Malawi, Nigeria, and Zambia, the VACS prompted respondents think of their most recent sexual partner from the past 12 months and report how often they used a condom with them; the same question was then asked regarding their second most recent and third most recent partners. In both cases, any reported lack of condom use was coded as 1, and otherwise as 0 (youth who had never had sex were also coded as 0). If any of the condom-related questions were missing data, it was coded as missing.

##### Multiple sexual partners

Number of sexual partners was a continuous variable (1.2% missing). We also created a binary version of this variable, where 0 represented zero or one sexual partner and 1 represented two or more (1.1% missing). We retained the continuous variable “number of sexual partners” to lend greater nuance to bivariate and post-hoc analyses.

##### Early sexual debut

This indicator was defined as a binary wherein 1 represented early sexual debut (i.e., sexual debut before 15 years old [14]) and 0 represented no early sexual debut (respondent had never had sex or had had first sex at age 15 years or later) (1.3% missing).

#### Parental care status

The VACS asked if respondents were currently living with their biological mother and the same for their biological father. The variable of parental care status had categories for living with both biological parents (the reference group), living with biological mother only, living with biological father only, and living with neither biological parent (0.3% missing).

#### Control variables

Included as control variables were gender (0 = male, 1 = female; 0% missing), country (reference group = Kenya; 0% missing), age (ranging from 13 to 17 years; 0% missing), orphan status, and a household assets score.

Orphan status was a categorical variable with four levels: non-orphan, paternal orphan, maternal orphan, and double orphan. We used a binary version of this variable where 0 = non-orphan and 1 = orphan of any type (1.9% missing).

We created the household assets score to serve as a proxy of respondents’ economic status. The assets questions in common across the five VACS were whether the household possessed the following belongings: electricity, a radio, a television, a phone, a refrigerator, a paraffin lamp (in the case of Malawi, a “Koloboyi or paraffin lamp”), a bicycle, and a car/truck. Cronbach’s alpha for possession of these eight assets ranged from α = .62 to α = .73 when run separately within each of the five countries and α = .65 in the full pooled sample. In Zambia and Nigeria, the paraffin lamp and bicycle items reduced the alpha value; in Kenya and Malawi, only paraffin lamp reduced the alpha value. In countries where paraffin lamp or bicycle did not reduce the alpha value, the item-rest correlation values of these items were still low compared to other items. Thus, we excluded paraffin lamp and bicycle from the scale.

Cronbach’s alpha for the final, six-item household assets composite ranged from α = .71 to α = .78 within each country sample; across the five-country dataset, α = .76. The final scale was a summed score where 1 represented the presence of an asset; the possible range of the scale was 0 to 6 (2.7% missing).

### Data analysis

We combined data from the five country surveys into one dataset consisting of *N* = 6770 observations of youth ages 13 to 17 years, which we analyzed in Stata 16 IC [32]. The VACS use complex sample designs with clustering, stratification, and sample weights. So that country surveys with large sample sizes would not overpower those with smaller sample sizes, we adjusted the sample weights such that each country would carry equal weight in the combined dataset.

To analyze bivariate relationships amongst categorical variables (parental care status, orphan status, gender, lack of condom use, early sexual debut), we conducted crosstabs and reported the design-based Rao-Scott correction of the Pearson chi-squared statistic, which accounts for complex survey design such as sampling and weight variability [33]. For bivariate associations amongst categorical variables (parental care status, gender) and continuous variables (number of sexual partners), we conducted ordinary least squares regressions to obtain *t* statistics, as appropriate for survey-weighted data.

We used logistic regression to explore relationships between parental care status and whether a youth engaged in any sexual risk behavior. We first ran a model with parental care status as the predictor of interest, and covariates gender, respondent age, household assets, and country (Model 1a). Second, we added orphan status as a predictor (Model 1b). We then examined both models with an interaction term between youth gender and parental care status.

As follow-up analyses, to explore the individual components of sexual risk behaviors as outcomes, we conducted either logistic regressions (for lack of condom use, Models 2a and 2b; and early sexual debut, Models 4a and 4b) or a negative binomial regression (number of sexual partners; Models 3a and 3b), using the same predictors. In all analyses, missingness was handled with listwise deletion.

## Results

Descriptive statistics are presented in Table 1. The mean age was 14.9 years (*SD* = 1.4 years); half were girls. About half of the sample lived with both parents, 20% lived in non-parental care, and the remaining lived with either their father or mother only. 18.6% of youth had engaged in one or more sexual risk behaviors. The most prevalent sexual risk behavior was early sexual debut (13.1%), followed by non-condom use (10.1%) and having multiple sexual partners (9.2%).

**Table 1:** Descriptive statistics

	Percent or Mean (SD)					
	Overall	Kenya	Malawi	Nigeria	Tanzania	Zambia
Binary sexual risk behaviors						
Multiple sexual partners***	9.2%	8.3%	14.3%	4.3%	7.0%	11.9%
Sex without condom***	10.1%	4.6%	11.5%	12.4%	9.3%	13.1%
Early sexual debut***	13.1%	9.5%	20.0%	10.1%	8.6%	17.1%
Any risk behavior***	18.6%	13.6%	24.5%	16.7%	13.3%	25.1%
Had engaged in sex***	22.3%	17.9%	27.2%	20.4%	16.0%	30.2%
No. of sexual partners^††^	0.4 (1.2)	0.3 (0.9)	0.6 (1.3)	0.3 (1.0)	0.3 (1.2)	0.6 (1.1)
Age at sexual debut^†††a^	13.3 (2.7)	13.2 (2.8)	12.8 (2.5)	13.8 (3.5)	13.3 (2.8)	13.5 (2.0)
Household assets^†††^	2.2 (1.7)	2.1 (1.5)	1.4 (1.3)	3.1 (2.1)	1.8 (1.9)	2.3 (1.4)
Parental care status***						
Both parents	55.5%	55.1%	50.4%	70.1%	48.5%	54.2%
Mother only	19.6%	24.6%	21.6%	9.7%	21.9%	19.8%
Father only	4.9%	3.6%	3.2%	5.0%	7.4%	5.4%
Neither parent	20.0%	16.7%	24.8%	15.2%	22.3%	20.6%
Orphan status**						
Non-orphan	78.8%	78.1%	77.2%	86.0%	77.3%	75.7%
Maternal orphan	4.6%	3.6%	5.8%	4.4%	4.1%	5.1%
Paternal orphan	13.2%	14.8%	13.3%	8.2%	15.2%	14.3%
Double orphan	3.4%	3.4%	3.6%	1.5%	3.3%	4.9%
No. of observations	6770	1292	1070	1840	1783	785
Weighted proportion	100%	20.3%	20.0%	19.1%	20.4%	20.2%

Chi squared test to compare distribution by country: **p < .01, ***p < .001

Simple regression to compare means by country: ^††^*p <* .01, ^†††^*p <* .001

^a^*Note*: Among respondents who have ever engaged in sex

### Bivariate statistics

Youth’s parental care status significantly differed by gender (*F* (2.89, 3487.31) = 8.76, *P <* .001) (Table 2); 23.8% of girls lived with neither parent, while only 16.1% of boys did. Girls had significantly fewer sexual partners (*t* (1205) = − 4.32, *p <* .001) than boys, and were less likely to have early sexual debut (*F* (1, 1205) = 17.16, *p <* .001), but did not differ from boys in lack of condom use (*F* (1, 1205) = 0.96, *p =* .33).

**Table 2:** Parental care status by gender

	Male	Female
Both parents	59.6%	51.4%
Mother only	19.2%	20.0%
Father only	5.1%	4.7%
Neither parent	16.1%	23.8%
Total	100%	100%

Design-based *F*(2.89, 3487.31) = 8.76, *p <* .001

About half of respondents in maternal care were non-orphans and half were paternal orphans (i.e., their fathers had died). For respondents in paternal care, most (63.2%) were not orphans, while about a third had a deceased mother. Half of respondents in non-parental care were not orphans (Table 3).

**Table 3:** Orphan status by parental care status

	Parental care status		
	Mother only	Father only	Neither parent
Non-orphan	50.4%	63.2%	49.5%
Paternal orphan	49.6%	–	19.1%
Maternal orphan	–	36.8%	14.2%
Double orphan	–	–	17.2%
Total	100%	100%	100%

Design-based *F*(6.25, 7532.74) = 224.88, *p <* .001

Condom use varied significantly by parental care status, *F* (2.98, 3589.29) = 27.05, *p <* .001, with youth living with no parents having the highest rate of non-condom use. Early sexual debut also varied significantly by parental care status, *F* (2.97, 3577.10) = 4.87, *p =* .002, with youth living with no parents having the highest rate of early sexual debut.

Ordinary least squares regression also showed that number of sexual partners varied significantly by parental care status, *F* (3, 1203) = 11.41, *p <* .001. Compared to youth living with both parents, youth living with no parents had significantly more sexual partners (*t* (1205) = 5.17, *p <* .001), while differences were not significant for youth living with only their father (*t* (1205) = − 1.23, *p =* .22) or only their mother (*t* (1205) = 1.60, *p =* .11).

### Multivariate statistics

Table 4 shows the logistic regression results for our main models. In Model 1a, living with neither parent (compared with both parents) was associated with increased odds of engaging in sexual risk behavior (*OR* = 2.26, 95% CI: [1.80, 2.83]). Girls had lower odds of engaging in sexual risk behavior than boys (*OR* = 0.73, 95% CI: [0.57, 0.94]).

**Table 4:** Logistic regression results for predicting presence of any sexual risk taking

	Odds Ratio (95% CI)	
	Model 1a	Model 1b
Parental care (ref = both parents)		
Mother only	0.96 (0.73, 1.26)	1.10 (0.82, 1.46)
Father only	1.07 (0.70, 1.63)	1.14 (0.73, 1.78)
Neither parent	2.26 (1.80, 2.83)***	2.56 (1.96, 3.33)***
Gender (ref = male)		
Female	0.73 (0.57, 0.94)*	0.73 (0.56, 0.94)*
Household assets	0.80 (0.75, 0.85)***	0.80 (0.75, 0.85)***
Age	1.51 (1.41, 1.63)***	1.53 (1.42, 1.65)***
Country (ref = Kenya)		
Malawi	1.79 (1.15, 2.79)*	1.77 (1.13, 2.77)*
Nigeria	1.68 (1.20, 2.35)**	1.63 (1.16, 2.30)**
Tanzania	0.86 (0.58, 1.27)	0.83 (0.55, 1.24)
Zambia	2.30 (1.62, 3.28)***	2.28 (1.59, 3.27)***
Orphanhood (ref = non-orphan)		
Orphan	–	0.83 (0.62, 1.12)
No. of observations	6423	6342
*F* statistic	26.91***	25.58***
*F* degrees of freedom	10, 1196	11, 1195

* *p* < .05, ** *p* < .01, *** *p* < .001

When controlling for orphan status (Model 1b), living with neither parent was still associated with increased odds of engaging in sexual risk behavior (*OR =* 2.56, 95% CI: [1.96, 3.33]), and being female with decreased odds (*OR =* 0.73, 95% CI: [0.56, 0.94]). Being an orphan was not significantly associated with the odds of engaging in sexual risk behavior.

We found no statistically significant interaction effects between gender and parental care status on the outcome of sexual risk behavior (data not shown).

#### Post-hoc models

We ran three post-hoc models to explore which underlying indicators were responsible for these dynamics (Table 5).

**Table 5:** Post-hoc regression analyses on indicators of sexual risk behavior

	Any lack of condom use (binary)^a^ Odds Ratio (95% CI)		Number of sexual partners (continuous)^b^ Coefficient (SE)		Early sexual debut (binary)^a^ Odds Ratio (95% CI)	
	Model 2a	Model 2b	Model 3a	Model 3b	Model 4a	Model 4b
Parental care (ref = both parents)						
Mother only	0.83 (0.58, 1.19)	1.01 (0.69, 1.49)	0.12 (0.13)	0.15 (0.16)	0.87 (0.64, 1.18)	0.98 (0.71, 1.34)
Father only	0.91 (0.50, 1.66)	1.01 (0.53, 1.91)	−0.32 (0.20)	− 0.32 (0.21)	1.07 (0.64, 1.77)	1.14 (0.66, 1.95)
Neither parent	2.86 (2.14, 3.81)***	3.35 (2.38, 4.72)***	0.61 (0.10)***	0.60 (0.12)***	1.60 (1.23, 2.08)***	1.80 (1.31, 2.46)***
Gender (ref = male)						
Female	1.03 (0.80, 1.34)	1.02 (0.79, 1.32)	−0.63 (0.12)***	−0.64 (0.12)***	0.51 (0.39, 0.68)***	0.51 (0.38, 0.68)***
Household assets	0.79 (0.73, 0.85)***	0.78 (0.73, 0.85)***	−0.14 (0.03)***	−0.14 (0.03)***	0.81 (0.75, 0.86)***	0.80 (0.75, 0.85)***
Age	1.62 (1.46, 1.79)***	1.62 (1.47, 1.80)***	0.45 (0.04)***	0.46 (0.04)***	1.11 (1.04, 1.19)**	1.12 (1.05, 1.20)**
Country (ref = Kenya)						
Malawi	2.35 (1.38, 4.00)**	2.30 (1.35, 3.94)**	0.57 (0.19)**	0.55 (0.19)**	1.98 (1.30, 3.01)**	1.95 (1.28, 2.98)**
Nigeria	4.05 (2.57, 6.40)***	3.90 (2.45, 6.21)***	0.15 (0.17)	0.07 (0.17)	1.29 (0.91, 1.83)	1.27 (0.89, 1.81)
Tanzania	1.78 (1.10, 2.88)*	1.70 (1.03, 2.79)*	−0.09 (0.20)	−0.12 (0.20)	0.85 (0.55, 1.30)	0.82 (0.53, 1.28)
Zambia	3.35 (2.09, 5.37)***	3.28 (2.03, 5.30)***	0.59 (0.17)**	0.56 (0.17)**	2.01 (1.45, 2.80)***	1.99 (1.43, 2.79)***
Orphanhood (ref = non-orphan)						
Orphan	–	0.74 (0.52, 1.04)	–	−0.02 (0.14)	–	0.82 (0.60, 1.14)
Constant	< 0.00 (< 0.00, < 0.00)	< 0.00 (< 0.00, < 0.00)	−7.69 (− 0.60)	−7.73 (0.60)	0.04 (0.01, 0.12)	0.04 (0.01, 0.11)
No. of observations	6544	6461	6452	6368	6443	6361
*F* statistic	24.01***	22.34***	32.47***	30.69***	14.03***	13.00***
*F* degrees of freedom	10, 1196	11, 1195	10, 1196	11, 1195	10, 1196	11, 1195

* *p* < .05, ** *p* < .01, *** *p* < .001

^a^ Logistic regression

^b^ Negative binomial regression

##### Lack of condom use

Youth living with no parents had statistically significantly higher odds of engaging in sex without a condom (*OR =* 2.86, 95% CI: [2.14, 3.81]; Model 2a) and this association remained after controlling for orphan status (*OR =* 3.35, 95% CI: [2.38, 4.72]); Model 2b). Orphanhood and gender were not associated with statistically significantly different odds of non-condom use.

##### Number of sexual partners

Non-parental care was associated with a higher number of sexual partners (*β =* .61, *p <* .001) regardless of gender, and girls had significantly fewer sexual partners than boys (*β =* −.63, *p <* .001; Model 3a). We find similar results after adding orphanhood as a covariate (Model 3b); moreover, orphanhood was not a significant predictor of the number of sexual partners.

##### Early sexual debut

1 Youth in non-parental care had higher odds of sexual debut before age 15 (*OR =* 1.60, 95% CI: [1.23, 2.08]), and girls also had lower odds (*OR =* 0.51, 95% CI: [0.39, 0.68]; Model 4a). We found similar results after controlling for orphanhood, and furthermore, orphanhood was not significantly associated with early sexual debut.

## Discussion

This study is the first to our knowledge that uses nationally-representative data to examine correlates of sexual risk taking and parental care status among youth in SSA. We found that 13- to 17-year-olds living without either biological parent had 156% increased odds of engaging in any sexual risk behaviors compared to those living with both parents, even while controlling for orphanhood, gender, poverty, and country differences.

Specifically, non-parental care was linked to heightened odds of non-condom use, sexual debut before age 15 years, and a higher number of sexual partners. These results expand upon findings that had linked orphanhood to having multiple sexual partners in Zimbabwean [34] and South African girls [27]. They reinforce findings from Ghana that link non-parental care with earlier age at sexual debut [20] and that link parental death with early sexual debut in both sexes [21, 22, 24, 25].

Our analyses found that girls were at lowered risk of engaging in any sexual risk behavior than boys. Boys had higher odds of early sexual debut and had more sexual partners, but did not have different odds of non-condom use. It is difficult to compare these findings to the existing literature on orphanhood, as statistical models usually look at just girls, or boys and girls separately, rather than controlling for orphanhood and gender in one model [21, 22, 24, 25, 34]. In Ghana, however, after controlling for parental care status and many other covariates, girls had significantly earlier ages of sexual debut than boys, contradicting our findings [20]. Our findings also failed to reinforce a study in South Africa that linked parental death to non-condom use in boys but not girls [21]. It is likely that other variables may explain the relationships among orphanhood, gender, and sexual risk, including perhaps parental care status. One review of literature in high-income countries suggests that parental monitoring is an important protective factors for boys with regards to sexual risk taking, while parental warmth is important for girls [18], but more research is necessary in order to elucidate these dynamics in the context of SSA.

Living with only a biological mother or only a biological father did not predict sexual risk behavior in our sample. This null relationship reinforces the Ghanaian study of parental care status, where neither paternal-only care nor maternal-only care was significantly linked to age at sexual debut [20], but contradicts a South African study that found being in maternal care delayed sexual debut amongst boys [35]. Our study also failed to reinforce findings that link maternal death to sexual activity and sexually transmitted infections [23], and non-condom use amongst girls [34].

This study has some important limitations. First, we were limited to using the variables available across all five countries, and there were slight differences in the wording of the survey questions about condom use that caution against making country comparisons of this outcome. Our analysis was limited to the five VACS currently available in SSA, which included four countries in East Africa and one in West Africa, so the data cannot be generalized to all of SSA. The data were collected in different years (from 2010 to 2014), and public policies or public health campaigns that happened during this time frame could have affected outcomes over time. Social desirability bias may have affected the data, as the surveys were conducted face to face and asked questions about sexual behavior. We could only look at parental care status of the youth at the time they were surveyed. The VACS do not capture parental care history or record other nuances in family composition (e.g., whether they lived with stepparents, in kinship care, or in foster care with unrelated caregivers). Most importantly, although this study attempts to elucidate the situation of children outside parental care, the VACS and other household surveys overlook children living outside of households, such as in residential care institutions, orphanages, or on the street. Such children are severely undercounted in official statistics and large-scale data collection efforts, a fact that children’s rights groups have entreated international organizations and governments to rectify [36]. However, this study is an important contribution to a field that usually only focuses on orphan status, while children may live without their parents regardless of whether or not their parents have died.

## Conclusion

This study is the first to our knowledge to link parental care status and sexual risk taking in a multi-country, nationally-representative analysis of Sub-Saharan African youth. Though prior research across the sub-continent had found consistent links between orphanhood and sexual risk taking, this analysis provides credence to the idea that it is not parental death specifically, but rather parental absence in general, that leads to sexual risk. Thus, it may be wise for youth HIV interventions in SSA to look beyond the conventional framing of “orphaned children” as at risk, instead considering all children who lack parental oversight as a potential intervention population within which to improve public and sexual health. Future research should expand on this investigation by looking at diverse caregiver arrangements, including care by grandparents, aunts and uncles, stepparents, and non-relatives, while including children living outside households in orphanages and institutions.

## Data Availability

The datasets analyzed during the current study are public-use and are available upon request from Together For Girls at https://www.togetherforgirls.org/request-access-vacs/

## Abbreviations

SSA: Sub-Saharan Africa
CDC: Centers for Disease Control and Prevention
VACS: Violence Against Children Survey
HIV: Human immunodeficiency virus
AIDS: Acquired immunodeficiency syndrome

## Footnotes

1 Since early sexual debut was defined as sex before age 15, as a robustness check, we also ran these analyses on respondents 15 to 17 only. Gender and non-parental care were still significantly associated with early sexual debut in these models.
